# 

**Published:** 1859-11

**Authors:** 


VOL. II.] PUBLISHED MONTHLY. [No. 11.

### THE CHICAGO MEDICAL JOURNAL.

EDITED BY

DANIEL BRAINARD, M. D.

Professor of Surgery in Rush Medical College; Surgeon to the United States Marine Hospital, the Chicago City Hospital, etc.

AND

W. GODFREY DYAS, M. D.,

Fellow of the Royal College of Surgeons, Ireland; late Demonstrator of Anatomy in the University of Dublin, etc.

ASSISTED BY EDWIN POWELL, M. D.

[v. 16]

NOVEMBER, 1859.

CHICAGO:

JAMES BARNET, BOOK AND JOB PRINTER, 189 LAKE STREET.

To whom all advertisements for insertion in the JOURNAL may be addressed.

AT TWO DOLLARS PER ANNUM, IN ADVANCE.

